# Improved disease markers suggest dual response in a patient with metastatic castration resistant prostate cancer and chronic lymphocytic leukemia following active cellular immunotherapy

**DOI:** 10.1186/s13045-015-0149-x

**Published:** 2015-05-14

**Authors:** Heather H. Cheng, Colleen Soleau, Evan Y. Yu

**Affiliations:** University of Washington, Seattle, USA; Fred Hutchinson Cancer Research Center, Seattle, USA; Division of Medical Oncology, Department of Medicine, University of Washington, Seattle Cancer Care Alliance, 825 Eastlake Avenue E, Seattle, WA 98109 USA

**Keywords:** Prostate cancer, Chronic lymphocytic leukemia (CLL), Sipuleucel-T, Immunotherapy, Prostate-specific antigen (PSA)

## Abstract

Prostate cancer and chronic lymphocytic leukemia (CLL) are relatively common malignancies associated with advanced age. Although immunotherapy-based strategies are used to treat both, currently, there is no overlap in specific therapies. Sipuleucel-T is an active cellular immunotherapy that improves overall survival for patients with metastatic castration resistant prostate cancer (mCRPC) but is not typically associated with a decline in prostate-specific antigen (PSA) following administration. We report the case of a 78-year-old man with mCRPC and Rai stage 0 CLL who sustained a 12-month decline in both PSA and white blood cell (WBC) count following treatment with APC8015-2 (an investigational form of sipuleucel-T), as part of the phase II ProACT clinical trial. Two years later, the patient received commercial sipuleucel-T and again was noted to have a decline in PSA. Exploratory analysis did not clearly identify any peripheral immune markers associated with response. This case report suggests that treatment with sipuleucel-T can rarely lead to PSA decline, may have dual activity against both prostate cancer and CLL, and that these findings warrant further investigation.

## Background

Prostate cancer is the most common non-skin malignancy affecting men and is responsible for the majority of cancer deaths in men in the United States. The first-line treatment of metastatic prostate cancer includes androgen deprivation therapy (ADT), which leads to a varying period of disease control. Eventually, the disease progresses despite ADT, representing the castration resistant disease state. Treatment options for metastatic castration resistant prostate cancer (mCRPC) in the past decade have expanded greatly, with five new agents recently approved by the Food and Drug Administration (FDA), including the first-in-class autologous cellular immunotherapy, sipuleucel-T (Provenge®) [1, 2].

Sipuleucel-T is produced by ex vivo activation of patient-derived peripheral blood mononuclear cells by incubation with a fusion protein, PA2024, comprised of granulocyte-macrophage colony-stimulating factor (GM-CSF) and prostatic acid phosphatase, which is expressed on over 95% of prostate cancers [3]. The resulting autologous product containing activated antigen-presenting cells, sipuleucel-T, is subsequently infused into the patient. The randomized phase III IMPACT clinical trial demonstrated a 4.1-month improvement in median overall survival in asymptomatic or minimally symptomatic men with mCRPC treated with sipuleucel-T compared to those treated with control and led to the FDA approval of sipuleucel-T in April 2010, representing the first approved immunotherapy for prostate cancer [1].

Chronic lymphocytic leukemia (CLL) is the most common leukemia diagnosed in the United States and Western world. CLL is a B cell chronic lymphoproliferative neoplasm that occupies one end of a single disease spectrum defined by the WHO with small lymphocytic lymphoma (SLL), a mature, peripheral B cell neoplasm, at the other end. Like prostate cancer, CLL is a disease associated with older age as well as with a highly variable clinical course ranging from indolent disease requiring no treatment to aggressive, lethal disease [4, 5]. The modified Rai and Binet systems of classification have been clinically useful in prognosis and therapeutic management. Therapy varies based on clinical, prognostic, and molecular features, and options include nucleoside analogs, monoclonal antibodies, chemotherapy, selective kinase inhibitors, and autologous and allogeneic immunotherapy [6]. More recently, promising antitumor effects have been observed using T cells containing chimeric antigen receptors (CAR) in patients with advanced CLL [7–9], providing compelling evidence that immune-based therapies may be effective in CLL.

We report the case of a patient with mCRPC who also had Rai stage 0 CLL. He was initially treated with APC8015-2 (an investigational form of sipuleucel-T) as part of a clinical trial and later with commercial sipuleucel-T.

## Case presentation

### Initial diagnosis and treatment

The patient was initially diagnosed with localized Gleason 7 prostate cancer in 1989, underwent radical prostatectomy in 1990, and received salvage radiation in 1994, followed by an undetectable prostate-specific antigen (PSA).

In December of 2005, the patient had an episode of gross hematuria and underwent transurethral resection of bladder tumor for presumed bladder cancer. He subsequently underwent cystectomy on March 8, 2006 with pathology unexpectedly showing metastatic prostate adenocarcinoma, Gleason 4+5. Post-cystectomy, PSA was 28.48 ng/mL on March 28, 2006, and he was started on ADT on May 2, 2006. He reached PSA nadir of 0.19 ng/mL in November 2006, which was followed by a slowly rising PSA, representing castration-resistant disease.

In November of 2008, 2 years after initiation of ADT, PSA rose to 7.35 ng/mL. He was then started on ketoconazole 400 mg PO daily, which was discontinued in April 2009 (PSA 3.93 ng/mL) due to diarrhea attributed to the ketoconazole. PSA declined to a nadir of 0.04 ng/mL in August 2009 and later began to rise again.

### Treatment on phase II ProACT trial

In June of 2010, the patient’s PSA increased to 9.08 ng/mL. Restaging with CT and bone scans revealed a new bone lesion in the right sixth rib. He was enrolled in the phase II treatment of *PRO*state Cancer with *A*ctive *C*ellular Immuno*T*herapy (ProACT) dosing study of sipuleucel-T (also referred to as APC8015) in men with mCRPC. In this study, eligible patients were randomized to receive either sipuleucel-T, manufactured using the FDA-approved standard 10 μg/mL concentration of PA2024, or a product manufactured in a similar manner to sipuleucel-T but using a lower concentration of PA2024: 5 μg/mL (APC8015-5) or 2 μg/mL (APC8015-2).

**Fig. 1:**
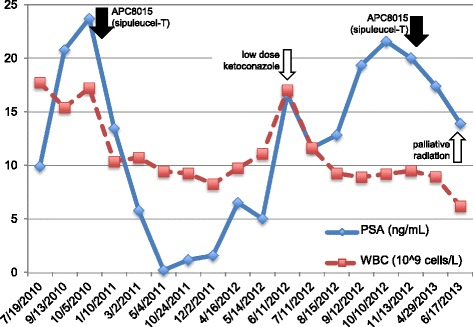
Pattern of PSA and WBC values following sipuleucel-T treatment. *Solid arrows*: initial APC8015-2 (investigational form of sipuleucel-T produced using 2 μg/mL of PA2024) in December 2010 and subsequent treatment with commercial sipuleucel-T (using 10 μg/mL of PA2024) in December 2012. *Open arrows*: other therapies. *Solid line*: *PSA* prostate-specific antigen; *Dotted line: WBC* white blood cell count

Of note, the patient had been diagnosed with Rai stage 0 chronic lymphocytic leukemia with a baseline white blood cell (WBC) count of 17.21 × 10^9^ cells/L and PSA of 23.68 ng/mL at time of starting study (Fig. 1).

The patient received three study infusions of study drug with the final dose administered on December 2, 2010. Subsequent unblinding revealed that he had been randomized to receive APC8015-2, the investigational form of sipuleucel-T produced using 2 μg/mL of PA2024. Correlative markers drawn for the ProACT study demonstrated that CD54 upregulation (a measure of product potency and antigen-presenting cell activation [1]) was low and overall immune responses were low to undetectable, as was cytokine production during the product manufacture (data not shown).

Approximately 6 months after study treatment, PSA had declined from 23.68 to a nadir of 0.25 ng/mL on May 4, 2011. In addition, WBC declined from 17.21 to 9.45 × 10^9^ cells/L with an absolute lymphocyte count (ALC) of 5.01 × 10^9^ cells/L, despite the fact that the patient was not receiving concurrent treatment for CLL; ALC was not consistently measured and is reported when available. This suggested that the APC8015-2 he received as part of the ProACT study rendered control of not only his prostate cancer but potentially also his CLL.

### Treatment with commercial sipuleucel-T

By April 16, 2012, the patient’s PSA, WBC, and ALC rose to 6.53, 9.75, and 5.55 ng/mL, respectively. He was subsequently retreated with commercial sipuleucel-T (produced using 10 μg/mL PA2024) with the final dose administered on December 31, 2012 (Fig. 1).

**Fig. 2:**
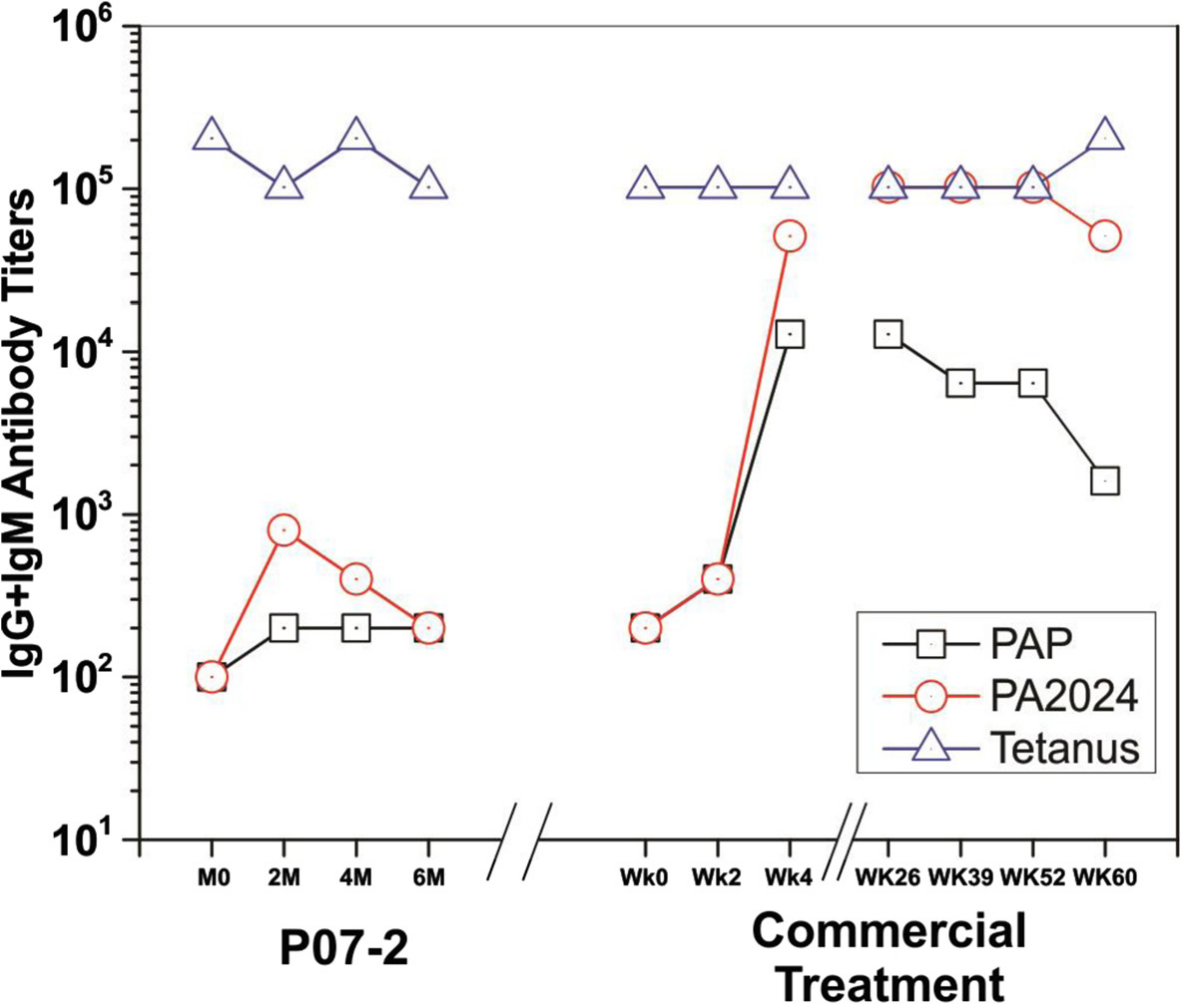
Pattern of IgG and IgM antibody titers following APC8015 treatment. Initial APC8015-2 (investigational form of sipuleucel-T produced using 2 μg/mL of PA2024) in December 2010 and with commercial sipuleucel-T (10 μg/mL PA2024) in December 2012. Rechallenge with commercial sipuleucel-T led to more robust IgG and IgM antibody titers. *PAP* prostatic acid phosphatase, *PA2024* fusion protein comprised of prostatic acid phosphatase and granulocyte-macrophage colony-stimulating factor

With Institutional Review Board (IRB) approval, the patient consented to undergo exploratory immune monitoring as part of his subsequent retreatment with commercial sipuleucel-T. Antigen-specific antibody responses (IgG and IgM) have previously been shown to correlate with survival after treatment [10]. Analysis of this patient’s samples showed a pronounced increase in antibody titers following commercial dose, though samples beyond week 6 were not collected (Fig. 2). T cell immune function against the prostate cancer antigens, PAP and PA2024, was measured using T cell proliferation and IFN-gamma (ELIspot) assays, product manufacturing culture media and serum cytokine profiling (eotaxin, IP-10, MCP-1, MCP-4, MIP-1β, TARC, IL-17, IL-1B, IL-6, TNF-α, IFN-γ, IL-10, IL-12 [p70], IL-13, IL-2, IL-4, and IL-5), as well as titers of antibodies hypothesized to cross-react with CLL antigens (e.g., CD19, CD20, CD23, CD38, bcl-2, cyclin D1, Zap-70) and CD54 levels (standard for product release parameters) did not reveal any clear patterns correlating with the observed PSA and WBC responses (data not shown).

### Subsequent disease course and treatment

Four months after commercial sipuleucel-T treatment, PSA had increased to 17.38 ng/mL and WBC was stable at 8.93 × 10^9^ cells/L on April 29, 2013. Unfortunately, the patient became more symptomatic from bone metastases and received palliative radiation to pubic rami and left acetabulum, completed in mid-June 2013. On June 17, 2013 PSA and WBC declined to 13.89 ng/mL and 6.2 × 10^9^ cells/L, respectively, likely reflecting radiation effect. Most recently, the patient has been treated with enzalutamide.

## Discussion

We report the case of an older patient with mCRPC and Rai stage 0 CLL who was treated with APC8015-2 (an investigational form of sipuleucel-T) and observed to have a dramatic and sustained PSA decline as well as improvement in his WBC. On subsequent treatment with commercial sipuleucel-T, he was also noted to have a PSA decline.

This patient’s initial PSA response to treatment was noteworthy since a PSA decline of 50 % from baseline was observed in only approximately 3 % of the participants in the phase III IMPACT trial [1], though PSA decline was observed at higher rates in the smaller phase I and II trials [11–13]. Moreover, following subsequent retreatment with commercial sipuleucel-T, his PSA declined again, albeit to a lesser degree and for a shorter duration (and palliative radiation 6 months post-treatment rendered subsequent PSA values problematic to interpret). Nevertheless, this pattern of PSA decline after treatment provides evidence that this patient was an exceptional responder to sipuleucel-T, although our exploratory analyses did not uncover a clear association with cytokine or immune markers.

A second feature in this patient’s case was his concurrent diagnosis of CLL and noted decline of WBC and ALC with study treatment, suggesting the possibility of cross-reacting antigens and dual therapeutic effect of sipuleucel-T against both mCRPC and CLL. During rechallenge with commercial sipuleucel-T, his WBC was in the normal range at the start of treatment, making it difficult to interpret the effect of rechallenge on CLL. Possible explanations include that either the WBC response following initial treatment was unrelated to sipuleucel-T or that the CLL response to the initial treatment of sipuleucel-T was more durable than the PSA response.

A recent report suggests that sipuleucel-T may induce humoral antigen spread [14]. It is possible that humoral antigen spread developed to include CLL antigens, though our exploratory immune monitoring, limited to a few known CLL antigens, did not support this. An alternative explanation is that CLL cells were likely pheresed along with normal leukocytes and CLL antigens were inadvertently introduced into the product processing in sufficient quantities to elicit response not only to PA2024 but also to CLL antigens.

Of note, active cellular immunotherapy is an approach being investigated in CLL [15], including one report in the literature of a dendritic cell vaccine in a small study of 15 patients using dendritic cells that endocytosed apoptotic bodies of CLL cells in combination with GM-CSF elicited anti-CLL immune responses though no clinical responses [16]. Another study of 22 patients with advanced CLL demonstrated that a vaccine consisting of irradiated autologous CLL cells admixed with GM-CSF-secreting cells administered after allogeneic hematopoetic transplantation resulted in CD8+ T cells consistently reacting against autologous tumor [17]. In addition, a prior study using the sipuleucel-T platform but targeted to multiple myeloma reported promising preliminary results [18]. Together with our case, these suggest that the active cellular immunotherapy approach used for sipuleucel-T may be efficacious when applied to other cancers with distinct antigens. We speculate that our patient may have had introduction of not only the PA2024 fusion protein but also the CLL cells during product manufacture and that his underlying immune system was intrinsically primed to respond to active cellular immunotherapy directed at both the intended target of prostate cancer cells and the bystander target of CLL cells.

In summary, this interesting case illustrates that the active cellular immunotherapy sipuleucel-T can result in dramatic and sustained PSA responses. In addition, treatment with sipuleucel-T can potentially lead to dual responses to other malignancies including CLL. We believe both of these remarkable findings warrant further investigation.

### Ethics, consent, and permissions

Ethics approval was provided by the University of Washington IRB. Written informed consent was obtained from the patient for publication of this case report and accompanying images. A copy of the written consent is available for review by the Editor-in-Chief of this journal.

## Abbreviations

ADT: Androgen deprivation therapy
ALC: Absolute lymphocyte count
CLL: Chronic lymphocytic cancer
mCRPC: Metastatic castration resistant prostate cancer
PSA: Prostate-specific antigen
WBC: White blood cell
